# Rotavirus nonstructural protein 1 antagonizes innate immune response by interacting with retinoic acid inducible gene I

**DOI:** 10.1186/1743-422X-8-526

**Published:** 2011-12-08

**Authors:** Lan Qin, Lili Ren, Zhuo Zhou, Xiaobo Lei, Lan Chen, Qinghua Xue, Xinlei Liu, Jianwei Wang, Tao Hung

**Affiliations:** 1 State Key Laboratory of Molecular Virology and Genetic Engineering, Institute of Pathogen Biology, Peking Union Medical College & Chinese Academy of Medical Sciences, # 9 Dong Dan San Tiao, Dongcheng District, Beijing 100730, P. R. China

**Keywords:** Rotavirus, Nonstructural protein 1, Interferon, Retinoic acid inducible gene I

## Abstract

**Background:**

The nonstructural protein 1 (NSP1) of rotavirus has been reported to block interferon (IFN) signaling by mediating proteasome-dependent degradation of IFN-regulatory factors (IRFs) and (or) the β-transducin repeat containing protein (β-TrCP). However, in addition to these targets, NSP1 may subvert innate immune responses via other mechanisms.

**Results:**

The NSP1 of rotavirus OSU strain as well as the IRF3 binding domain truncated NSP1 of rotavirus SA11 strain are unable to degrade IRFs, but can still inhibit host IFN response, indicating that NSP1 may target alternative host factor(s) other than IRFs. Overexpression of NSP1 can block IFN-β promoter activation induced by the retinoic acid inducible gene I (RIG-I), but does not inhibit IFN-β activation induced by the mitochondrial antiviral-signaling protein (MAVS), indicating that NSP1 may target RIG-I. Immunoprecipitation experiments show that NSP1 interacts with RIG-I independent of IRF3 binding domain. In addition, NSP1 induces down-regulation of RIG-I in a proteasome-independent way.

**Conclusions:**

Our findings demonstrate that inhibition of RIG-I mediated type I IFN responses by NSP1 may contribute to the immune evasion of rotavirus.

## Background

Rotavirus is a major cause of acute diarrhea in children under 5 years old, leading to approximately 600,000 annual deaths in the world [1]. Although two live vaccines, an attenuated human rotavirus strain (Rotarix™) and a pentavalent human-bovine reassortant (Rotateq™), have been demonstrated to protect recipients from rotavirus infection effectively and safely in clinical trials and have been licensed in several countries, the protective mechanisms of rotavirus vaccines and the pathogenic mechanisms of rotavirus are not fully understood [2,3]. A better understanding of the pathogenic mechanisms of rotavirus infection, especially how rotaviruses subvert and evade host antiviral responses are essential for identifying novel strategies to develop antiviral reagents and new vaccines.

The type I interferon (IFN) mediated immune response constitutes the first line of host defense against virus infection [4]. Host cells respond to viral infection by producing IFNs, which further trigger the expression of a variety of genes involved in antiviral responses through the Janus Kinase/Signal Transducer and Activator of Transcription (JAK/STAT) pathway [5]. IFNs also stimulate downstream immune events, leading to the activation of specific immune cells involved in adaptive immune responses [6,7]. To counteract antiviral responses induced by IFN-α/β, most viruses have evolved viral products to suppress the IFN-mediated signaling pathways [8]. For example, NS1 of influenza virus, NS1/NS2 of respiratory syncytial virus (RSV), VP35 of Ebola virus, E6 protein of human papilloma virus (HPV), and 3C of enterovirus 71 suppress IFN induction by inhibiting IFN signaling pathways [9-14].

Rotaviruses, members of the *Reoviridae *family, are non-enveloped icosahedra viruses containing 11 segments of a double stranded RNA (dsRNA) genome within a triple-layered particle. The rotavirus genome encodes six structural proteins (VPs) and six nonstructural proteins (NSPs). The structural proteins (VP1-4, VP6-7) form the virion. The NSPs (NSP1-6) function in dsRNA replication, transcription and translation of viral mRNA, and maturation of viral particles [1]. Rotavirus NSP1, a 55-kDa RNA binding protein, is the product of the rotavirus gene 5. It has been shown that the interaction between NSP1 and host signaling proteins is essential for rotaviruses to subvert innate immune responses. NSP1 inhibits innate immune signaling by the following mechanisms. First, NSP1 induces proteasome-dependent degradation of the interferon transcription factors (IRF3, IRF7, and IRF5) to inhibit the IFN response [15-17]. Second, NSP1 inhibits nuclear factor-κB (NF-κB) activation by inducing proteasome-dependent degradation of β-transducin repeat containing protein (β-TrCP) and subsequent IFN-β gene transcription [18]. Third, rotavirus efficiently antagonizes cellular antivirus responses by preventing the nuclear accumulation of STAT1, STAT2, and NF-κB [19].

NSP1 is the least conserved protein among rotavirus strains [20]. The effect of NSP1 on innate immunity appears rotavirus strain-specific [21]. Investigations on the NSP1 proteins of different rotavirus strains have shown that some degrade IRFs, some degrade β-TrCP, and some target both [21]. For instance, the porcine OSU strain NSP1 cannot induce IRF3 degradation, but it induces the degradation of β-TrCP [21]. We hypothesize that, aside from IRFs and β-TrCP, NSP1 might target other cellular substrates involved in antiviral signaling pathways.

In this study, we investigated whether NSP1 targets other proteins involved in IFN response. We found that NSP1 can inhibit virus-induced activation of IFN-β promoter independent of IRF3 degradation. Furthermore, we show that retinoic acid inducible gene I (RIG-I)-mediated induction of IFN-β is inhibited by NSP1. Our study also revealed that NSP1 interacts with RIG-I and mediates RIG-I down-regulation in a proteasome-independent way. Thus, RIG-I may be an additional target that is antagonized by rotavirus NSP1.

## Results

### Rotavirus NSP1 inhibits IFN-β promoter activation independent of IRF3 degradation

Previous studies have shown that the NSP1 protein of the simian rotavirus SA11 strain subverts host innate immune response by inducing degradation of IRF family proteins [16,17]. NSP1 interacts with IRF3 through its C terminal IRF3 binding domain [15,17]. However, research on the porcine rotavirus OSU demonstrated that OSU NSP1 bound weakly to IRF3 and did not cause IRF3 degradation. This observation suggested the possibility of alternative targets for NSP1 in counteracting antiviral responses.

**Figure 1:**
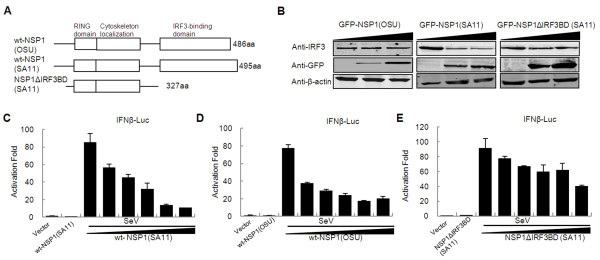
**Rotavirus NSP1 inhibits IFN-β promoter activation independent of IRF3 degradation**. (A) Scheme of full-length (wt) NSP1 structures of rotavirus OSU and SA11 strains and C-truncated (ΔIRF3 binding domain) NSP1 mutant of rotavirus SA11. (B) Western blot analysis for degradation of IRF3 by OSU NSP1, SA11 NSP1 and NSP1ΔIRF3BD (SA11). 293FT cells were transfected with pCMV-IRF3, pEGFP-OSU NSP1 or pEGFP-SA11 NSP1 or pEGFP-NSP1ΔIRF3BD (SA11) plasmids and cell extracts were assayed 48 h post-transfection for the expression of IRF3. Immunoblots were probed with anti-IRF3 monoclonal antibody (top panel). β-actin was used as a loading control (bottom panel). (C, D, E) Inhibition of virus-induced IFN-β promoter activation by SA11 NSP1 (C), OSU NSP1 (D) or NSP1ΔIRF3BD (SA11) (E). 293FT cells were transfected with pGL3-IFN-β-Luc, pRL-SV40, and increasing amounts of SA11 NSP1, OSU NSP1 or NSP1ΔIRF3BD (SA11) expression plasmids. Cells were infected with Sendai virus for 24 h and assayed for luciferase activities. Data are expressed as folds of activation with standard deviations among triplicate samples.

To investigate whether NSP1 targets other proteins involved in IFN response, we tested whether NSP1 could inhibit virus-induced IFN-β promoter activation in an IRF3 degradation-independent way. For this purpose, we made NSP1 constructs expressing wild type OSU NSP1 and an IRF3 binding domain truncated SA11 NSP1 (NSP1ΔIRF3BD) (Figure 1A), and then tested the ability of these constructs to mediate IRF3 degradation in 293FT cells. We found that unlike SA11 NSP1, both OSU NSP1 and SA11 NSP1ΔIRF3BD were unable to induce the degradation of IRF3 (Figure 1B). We then evaluated whether OSU NSP1 and SA11 NSP1ΔIRF3BD could inhibit virus induced IFN-β promoter activity by transfecting 293FT cells with an IFN-β luciferase reporter plasmid along with the OSU NSP1 or SA11 NSP1ΔIRF3BD construct. After transfection, cells were stimulated with Sendai virus and were then lysed for luciferase assays. Our results indicate that OSU NSP1 and SA11 NSP1 significantly suppress the promoter activity of IFN-β in a dose-dependent manner (Figure 1C and 1D). Although a little weaker than wild type NSP1, SA11 NSP1ΔIRF3BD still inhibited IFN-β promoter activity in a dose-dependent manner (Figure 1E). These results suggest the existence of an alternative target for NSP1-mediated IFN pathway inhibition other than IRF3.

### NSP1 inhibits RIG-I mediated IFN-β promoter activation

**Figure 2:**
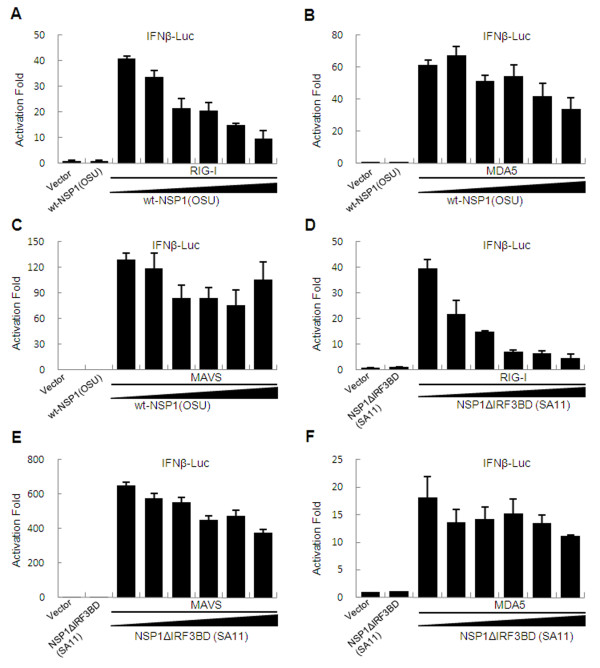
**Rotavirus NSP1 inhibits RIG-I mediated IFN-β promoter activation**. 293FT cells were transfected with pGL3-IFN-β-Luc along with plasmids encoding RIG-I (A and D), MDA5 (B and F), MAVS (C and E) and OSU NSP1 or SA11 NSP1ΔIRF3BD. pRL-SV40 was included as a control. At 36 h after transfection, the luciferase activities were measured as described in the Methods section. Results are expressed as folds of activation with standard deviations among triplicate samples.

To investigate further the potential host target of NSP1, we tested the inhibition effects of NSP1 on IFN-β promoter activation induced by several key innate immune signaling proteins upstream of IRF3, including RIG-I, melanoma differentiation-associated gene 5 (MDA5), and the mitochondrial antiviral-signaling protein (MAVS, also known as IPS-1/VISA/Cardif). Notably, RIG-I-mediated IFN-β activity was strongly inhibited by OSU NSP1 and SA11 NSP1ΔIRF3BD in a dose-dependent manner (Figure 2A and 2D); whereas, MDA5, another helicase for RNA virus recognition other than RIG-I, and MAVS, the downstream adaptor molecule for RIG-I, were fully competent to induce IFN activity in the presence of OSU NSP1 and SA11 NSP1ΔIRF3BD (Figure 2B, C, E and 2F). Taken together, these findings indicate that RIG-I could be a potential target for NSP1.

### NSP1 interacts with RIG-I

**Figure 3:**
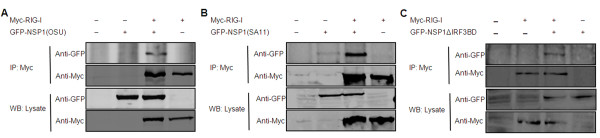
**Analysis of interaction between NSP1 and RIG-I**. (A) Immunoprecipitation analysis for the association between OSU NSP1 and RIG-I. 293FT cells were transfected with plasmids encoding full-length Myc-RIG-I, GFP, and GFP-OSU NSP1. At 48 h after transfection, cell lysates were immunoprecipitated (IP) with antibodies against Myc, and detected by SDS-PAGE. Immunoprecipitates and aliquots of cell lysates were then assayed by Western blot (WB) analysis. (B) SA11 NSP1 interaction with RIG-I. 293FT cells were transfected with plasmids encoding Myc-RIG-I, GFP-SA11 NSP1, and GFP separately. Immunoprecipitation and Western blot analysis were performed as described for panel A. (C) Interaction between NSP1ΔIRF3BD of rotavirus SA11 and RIG-I. Transfection, immunoprecipitation and Western blot analysis were performed as described above.

Subsequently, we examined whether NSP1 can interact with RIG-I. We cotransfected the OSU NSP1 and RIG-I constructs into 293FT cells and performed immunoprecipitation analysis using antibodies specific to Myc (RIG-I). Our findings show that OSU NSP1 coprecipitates with RIG-I by anti-Myc antibody (Figure 3A). We further tested the potential interaction between SA11 NSP1 and RIG-I, and immunoprecipitation analysis suggested that SA11 NSP1 could also interact with RIG-I (Figure 3B).

Given that OSU NSP1 is unable to degrade IRF3, unlike SA11 NSP1, and that both OSU NSP1 and SA11 NSP1 can interact with RIG-I, we questioned if IRF3 binding domain is involved in NSP1-RIG-I interaction. SA11 NSP1ΔIRF3BD and RIG-I were cotransfected into 293FT cells, and immunoprecipitation analysis was performed using antibodies specific to the Myc (RIG-I). We found that SA11 NSP1ΔIRF3BD can interact with RIG-I (Figure 3C), indicating that N-terminal of SA11 NSP1 protein is involved in NSP1-RIG-I interaction, whereas the IRF3 binding domain of NSP1 may not be involved.

Collectively, these data suggest that NSP1 protein of different rotavirus strains may be associated with RIG-I. Whether or not this interaction between NSP1 and RIG-I is due to direct binding or through a protein complex remains to be elucidated.

### RIG-I is down-regulated at protein level by NSP1 but is proteasome-independent

**Figure 4:**
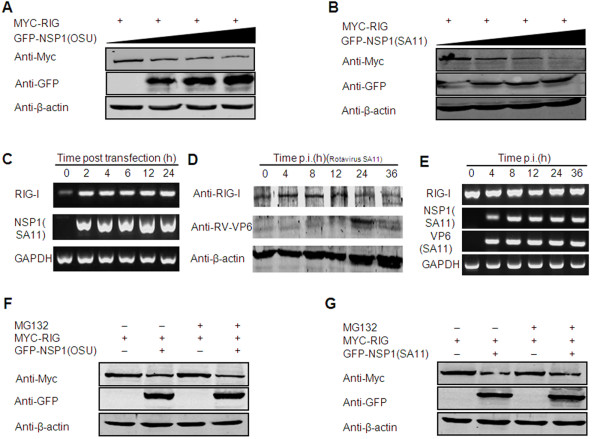
**RIG-I is down-regulated by NSP1 at the protein level but is proteasome-independent**. (A, B) Western blot analysis of RIG-I down-regulation by NSP1. 293FT cells were transfected with increased amount of pEGFP-OSU NSP1 (A) or pEGFP-SA11 NSP1 (B) and plasmid encoding Myc-RIG-I. Cell extracts were prepared 48 h post-transfection. Immunoblots were probed with anti-Myc monoclonal antibody to detect RIG-I (top panel). β-actin was used as a loading control (bottom panel). (C) Transcription level of RIG-I at different time points after transfection. 293FT cells were co-transfected with SA11-NSP1 and RIG-I plasmids. At different time points after transfection, total RNA extracted from cells was subjected to RT-PCR amplification and electrophoresis for RIG-I, NSP1 and GAPDH (inner control) mRNAs. (D, E) RIG-I is degraded in rotavirus infected cells. MA104 cells were infected with rotavirus SA11 at a m.o.i. of 0.1. Cell extracts were prepared at 0, 4, 8, 12, 24 and 36 h post-infection (p.i). RIG-I protein levels at each time point p.i. were determined by Western blot analyses using an anti-RIG-I antibody. The viral protein VP6 was used as an indicator for rotavirus infection. β-actin was used as a loading control. RIG-I, NSP1, VP6 and GAPDH mRNAs were also checked in parallel for evaluating the transcription level (E). (F, G) Effects of a proteasome inhibitor on NSP1 mediated RIG-I down-regulation. 293FT cells were transfected with Myc-RIG-I, pEGFP-OSU NSP1 (F) or pEGFP-SA11 NSP1 (G). The cells were treated with the proteasome inhibitor MG132 or an equivalent volume of DMSO as described in Methods. Lysates were prepared 36-48 h post-transfection. Immunoblots were probed with anti-Myc to detect myc-tagged RIG-I.

Previous studies have demonstrated that NSP1 could inhibit the IFN-mediated antiviral response through inducing proteasome-dependent degradation of the interferon transcription factors (IRF3, IRF7, and IRF5) [8-10]. Because we found NSP1 could bind to RIG-I, we further investigated whether NSP1 could mediate RIG-I down regulation. To address these possibilities, 293FT cells were co-transfected with OSU NSP1 or SA11 NSP1 along with RIG-I. Lysates were prepared at 48 h post transfection and were analyzed by Western blot. We found that an increased expression of OSU NSP1 or SA11 NSP1 correlated well with a decreased expression of RIG (Figure 4A and 4B), suggesting that NSP1 may induce the down-regulation of RIG-I.

To detect if down-regulation of RIG-I protein is related to inhibition of RNA transcription, RIG-I was co-expressed with NSP1, and the mRNA level of RIG-I was monitored by RT-PCR at different time points post transfection. As shown in Figure 4C, the transcription of RIG-I was not reduced when NSP1 was co-expressed, indicating that the reduction of RIG-I protein levels is not a result of transcription inhibition. Further, we detected the levels of RIG-I protein in rotavirus infected cells. Of more physiological relevance, our results showed that protein levels of RIG-I were decreased upon rotavirus infection, whereas the mRNA level was not attenuated (Figure 4D and 4E). These findings indicate that the down-regulation of RIG-I by NSP1 is achieved at the protein level.

As it appears that NSP1 induces the proteasome-mediated degradation of targeted proteins [16-18], we tested whether NSP1 can mediate RIG-I down-regulation by proteasome. However, proteasome inhibition assays revealed that the proteasome inhibitor MG132 could not inhibit NSP1 induced RIG-I down-regulation (Figure 4F and 4G), indicating that proteasome-dependent proteolysis may not be involved in this process.

## Discussion

Previous studies revealed that rotavirus NSP1 may antagonize IFN-β signaling to support rotavirus replication [22,23]. Studies on the NSP1 proteins of several RV strains have shown that the degradation targets could be IRFs or/and β-TrCP [15-18,21]. In the present study, we found NSP1 protein of rotavirus OSU strain inhibits virus-induced activation of IFN-β promoter independent of IRF3 degradation (Figure 1), in agreement with previous reports [18,20]. In addition, we observed that the truncated NSP1 of the SA11 strain lacking the IRF3 binding domain (SA11 NSP1ΔIRF3BD) reserve its ability to inhibit virus-induced IFN-β promoter activity (Figure 1), indicating that other targets may exist by which NSP1 inhibits the IFN response.

RIG-I is an intracellular molecule that responds to viral nucleic acids and activates downstream signaling involved in innate immune responses. RIG-1 induces several members of type I interferon (IFN) family, which consist of the most important effectors of the innate antiviral immune system [24]. Accumulating evidence demonstrates that RIG-I is a key component in antiviral immune responses [25]. The production of IFN was abrogated in conventional dendritic cells (cDCs) from RIG-I^-/- ^mice infected with Newcastle disease virus, Sendai virus, and vesicular stomatitis virus (VSV) [26], indicating that RIG-I plays a pivotal role in sensing RNA virus infections. Recently, some experiments assessed the importance of RIG-I in rotavirus infection. Rotavirus infection-induced IFN-β secretion and interferon stimulated response element (ISRE) activation were impaired by silencing RIG-I, but not by silencing toll-like receptor 3 (TLR3) or protein kinase R (PKR). Furthermore, rotavirus replication was increased in RIG-I depleted cells [27], indicating that RIG-I may be critical for combating rotavirus infection [28,29].

It is not surprising that viruses have evolved a variety of mechanisms to counteract RIG-I-mediated signaling. Some viruses, such as the Ebola virus, encode proteins that bind dsRNA to prevent their detection by RIG-I and MDA-5 [30]. Some other viruses, such as rhinovirus, echovirus, and encephalomyocarditis virus, encode proteases that cleave RIG-I and attenuate RIG-I mediated IFN signaling [31,32]. Recently, our lab reported that the 3C protein of Enterovirus 71 suppresses RIG-I signaling by disrupting the RIG-I-MAVS complex [13]. Here we demonstrated that the non-structural protein of rotavirus, NSP1, could interact with RIG-I. Furthermore, NSP1 could promote RIG-I down-regulation in a proteasome-independent way, thus attenuating a RIG-I mediated immune response. These results suggest that RIG-I could be a novel host factor antagonized by rotavirus NSP1.

Some reports demonstrate that rotavirus NSP1 is an E3 ubiquitin ligase [33,34]. NSP1 may induce the proteasome-mediated degradation of targeted proteins [16-18]. This degradation raises the possibility that NSP1 mediated RIG-I down-regulation may be proteasome-dependent. However, here we show that the proteasome inhibitor MG132 did not block NSP1-induced RIG-I down-regulation (Figure 4). The underlying mechanism for this phenomenon is unknown. Further experiments, such as investigating if lysosome or caspase inhibitors could block the NSP1-induced RIG-I down-regulation, will provide insights into the detailed mechanisms of NSP1-host interactions.

**Figure 5:**
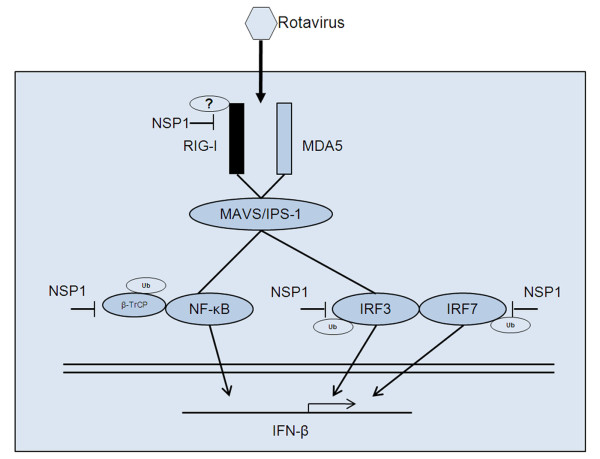
**Schematic diagram of the proposed mechanisms by which rotavirus NSP1 subverts host innate immunity**. Shown are the targets of NSP1 involved in host innate immune signaling. NSP1, nonstructural protein 1 of rotavirus; RIG-I, retinoic acid inducible gene I;MDA5, melanoma differentiation-associated gene 5;MAVS, mitochondrial antiviral-signaling protein; IPS-1, IFNβ-promoter stimulator 1; IFN, interferon; IRF, interferon transcription factor; Ub, ubiquitin.

Based on our findings and those of earlier studies by others [15-18,21,27-29], we can present an updated schematic diagram to show the proposed mechanism by which rotavirus NSP1 subverts host innate immunity (Figure 5). Rotaviruses were detected by RIG-I and/or MDA-5 when they enter host cells. Then RIG-I and/or MDA5 associate with MAVS/IPS-1, and antiviral signals are propagated to IRF3 and NF-κB. Rotavirus NSP1 mediates proteasomal degradation of IRF3 and IRF7 to inhibit the antiviral response and IFN-β secretion [16,17]. It is possible that NSP1 of some rotavirus strains mediates proteasomal degradation of β-TrCP, leading to inhibition of NF-κB [18]. It is also possible that NSP1 mediates the down-regulation of RIG-I to subvert host innate immune response.

## Conclusions

This study demonstrated that the NSP1 protein of rotavirus may interact with RIG-I and mediate down-regulation of RIG-I, thus interfering with the RIG-I-mediated signaling pathway. This activity is unrelated to its IRF3 binding ability. Our findings provide insight into the pathogenesis of rotavirus infection and new knowledge in the path to find novel targets for anti-viral therapies.

## Methods

### Cells and viruses

Human embryo kidney cells 293FT (Invitrogen, Carlsbad, CA) were cultured in Dulbecco's modified Eagle's medium (DMEM; Invitrogen) supplemented with 10% fetal bovine serum (FBS; HyClone, Logan, UT), 100 U/ml penicillin, and 100 μg/ml streptomycin at 37°C in a 5% CO_2 _humidified atmosphere. African green monkey kidney MA104 cells (ATCC, Manassas, VA) were maintained in DMEM supplemented with 5% FBS, 100 U/ml penicillin, and 100 μg/ml streptomycin. Rotavirus SA11 strain (CDC, USA) was propagated and titrated in MA104 cells. Sendai virus (SeV) was kindly provided by Professor Zhendong Zhao (Institute of Pathogen Biology, Chinese Academy of Medical Sciences, China).

### Plasmids

The full-length NSP1 open reading frame (ORF) of the rotavirus strain OSU (GenBank accession number U08432) was artificially synthesized and inserted into the entry vector pUC57 and the sequence was verified by Sangon Biotech (Shanghai, China). The OSU NSP1 cDNA was then transferred into the destination vector pEGFP-C1 (Clontech, Mountain View, CA). A vector containing the NSP1 ORF of the rotavirus SA11 strain (GenBank accession number AF290883) was prepared by PCR amplification of a plasmid that contains the SA11 gene 5 cDNA. The PCR product was digested with applicable restriction enzymes and inserted into the entry vector pCDNAII (Invitrogen) and then the insert was transferred into pEGFP-C1. NSP1 cDNA fragments encoding truncated forms of the IRF3 binding domain at C-terminal residues (SA11 NSP1ΔIRF3 BD) were cloned by PCR with the designed forward and reverse primers. The PCR products were ligated into the sites of vector pEGFP-C1. Expression constructs for retinoic acid inducible gene I (RIG-I), melanoma differentiation-associated gene 5 (MDA5), and mitochondrial antiviral-signaling protein (MAVS) were kindly provided by Dr. Bin He (University of Illinois at Chicago, USA). The IRF3 expressing plasmid pCMV6-XL4-IRF3 was a product from Origene Technologies (Rockville, MD). The pRL-SV40 and pGL3-IFN-β-Luc plasmids were generous gifts from Professor Zhendong Zhao.

### Transfection

293FT cells were cultured to approximately 70-80% confluence in 24-well plates, 10 cm dishes or 6-well plates and transfected with indicated plasmids using Lipofectamine 2000 (Invitrogen). After 24 or 48 h, cells were harvested and lysed in RIPA buffer [150 mM NaCl, 25 mM Tris (pH 7.4), 1% NP-40, 0.25% sodium deoxycholate, proteinase inhibitor cocktail tablets (Roche, Indianapolis, IN), and 1 mM EDTA]. In experiments examining the effect of the proteasome inhibitor MG132 (Calbiochem, Darmstadt, Germany), the culture medium was removed at different time points post-transfection (p.t.) and replaced with fresh medium containing 25 μM MG132. Cells were harvested at 36 or 48 h p.t. by lysis in RIPA buffer. For RIG-I mRNA detection, total cellular RNA was extracted using Trizol reagent (Invitrogen) at different time points after transfection.

### Rotavirus infection

The rotavirus SA11 strain was activated by incubation with acetylated trypsin (10 μg/ml) for 30 min at 37°C prior to infection. Approximately 2.5 × 10^5 ^MA104 cells in 6-well plates were infected at a multiplicity of infection (MOI) of 0.1 with activated rotavirus SA11. Virus was added to the cells for adsorption for 45 min at 37°C and then washed with media to remove unbound virus. At different time points post-infection (p.i.), cells were lysed in RIPA buffer and assayed for protein content by immunoblot analysis. Total cellular RNA was extracted simultaneously.

### Luciferase reporter assay

For the luciferase reporter assays, 293FT cells were cultured in 24-well plates at a cell density of 1.5 × 10^5 ^cells per well. Twelve hours later, cells were transfected with a control plasmid or plasmids expressing RIG-I, MDA5, or MAVS, and OSU NSP1 or SA11 NSP1ΔIRF3 BD along with pGL3-IFN-β-Luc and pRL-SV40 using Lipofectamine 2000. The total amount of DNA was kept consistent for all constructs by adding vector plasmids without inserts. Each sample was collected in triplicate, and each experiment was performed three times. At 48 h after transfection, cells were harvested, and cell lysates were used to determine luciferase activities using the Dual Luciferase Reporter Assay Kit (Promega, Madison, WI). Luciferase activity was normalized using *Renilla *luciferase as an internal control, and the fold induction of luciferase activity above control was calculated.

### Immunoprecipitation

Cells were collected at 48 h after cotransfection and then lysed with 25 mM Tris-HCl buffer (pH 7.4) containing 150 mM NaCl, 1% NP-40, 0.25% sodium deoxycholate, and proteinase inhibitor cocktail (Roche, Indianapolis, IN). Cell lysates were incubated overnight at 4°C with monoclonal antibodies against Myc (Sigma, St. Louis, MO) in the presence of protein A/G agarose beads (Santa Cruz Biotechnology, Santa Cruz, CA). Immunocomplexes captured on the affinity gel or protein A/G agarose beads were extensively washed with lysis buffer and eluted with SDS loading buffer by boiling for 5 min. Then the samples were subjected to SDS-PAGE and Western blot analysis.

### Reverse transcription-polymerase chain reaction (RT-PCR)

RNA samples were treated with DNase I (Pierce, Rockford, IL), and reverse transcription was carried out using a Superscript cDNA Synthesis Kit (Invitrogen) according to the manufacturer's instructions. cDNA samples were subjected to PCR amplification and electrophoresis to detect RIG-I and NSP1 expression. Specific primers for RIG-I (RIG-I-F-5'-CTCCCGGCACAGAAGTGT-3' RIG-I-R-5'-CCTCTGCCTCTGGTTTGG-3'), SA11-NSP1 (NSP1-F-5'-CATCTAATCACCCAGGCAATG-3 'NSP1-R-5'-TCACGAATCCGCCAATCA-3'), SA11-VP6 (VP6-F-5'-GACCAGTCTTTCCACCAGG-3' VP6-R-5'-GCCACTGTAAATATGCGTTG-3') and GAPDH (F-5'-GAAGGTGAAGGTCGGAGTC'-3' R-5'-GAAGATGGTGATGGGATTTC-3') were used.

### Western blot analysis

Cells were pelleted by centrifugation and lysed in RIPA buffer. Aliquots of cell lysates were resolved on 12% SDS-PAGE and transferred to a nitrocellulose membrane (Pall, Port Washington, NY). The membranes were blocked with 5% nonfat milk and then probed with anti-GFP monoclonal antibody (Sigma), anti-Myc monoclonal antibody (Sigma), anti-IRF3 monoclonal antibody (Santa Cruz Biotechnology), anti-RIG-I monoclonal antibody (Santa Cruz Biotechnology), anti-rotavirus VP6 monoclonal antibody (prepared in our laboratory) and anti-β-actin monoclonal antibody (Sigma) primary antibodies at 4°C overnight, respectively. This was followed by incubation with the corresponding IRD Fluor 800-labeled IgG or IRD Fluor 680-labeled IgG secondary antibody (Li-Cor, Lincoln, NE). After washing, the membranes were scanned using an Odyssey Infrared Imaging System (Li-Cor) at a wavelength of 700-800 nm and analyzed with Odyssey software. The molecular sizes of the developed proteins were determined by comparison with prestained protein markers (Fermentas, Maryland, CA).

## List of abbreviations

NSP1: nonstructural protein 1; IFN: interferon; IRF: IFN-regulatory factor; β-TrCP: β-transducin repeat containing protein; RIG-I: retinoic acid inducible gene I; MAVS: mitochondrial antiviral-signaling protein; JAK: Janus Kinase; STAT: Signal Transducer and Activator of Transcription; RSV: respiratory syncytial virus; HPV: human papilloma virus; dsRNA: double stranded RNA; NF-κB: nuclear factor-κB; NSP1ΔIRF3BD: an IRF3 binding domain truncated NSP1; MDA5: melanoma differentiation-associated gene 5; cDCs: conventional dendritic cells; VSV: vesicular stomatitis virus; ISRE: interferon stimulated response element; TLR3: toll-like receptor 3; PKR: protein kinase R; SeV: Sendai virus; ORF: open reading frame; MOI: multiplicity of infection; RT-PCR: reverse transcription-polymerase chain reaction.

## Competing interests

The authors declare that they have no competing interests.

## Authors' contributions

LQ, LR, ZZ, XL, LC, QX and ZS carried out the experiments. LQ, LR, ZZ, JW and TH designed the research, wrote the manuscript and reviewed the drafts. All authors read and approved the final manuscript.
